# Glances at the Hospitals

**Published:** 1905-03-04

**Authors:** 


### GLANCES AT THE HOSPITALS.

### BIRKENHEAD BOROUGH HOSPITAL.

THE total receipts amounted to £3,966, being £166 higher than in the preceding year, but the total fell short of the expenditure, which was £4,440. At the beginning of the year there was an adverse balance of £236, and this has now been increased to £709. The Committee remark that the income is not sufficient for the unavoidable outlay; but the annual subscriptions have slightly increased, and the work-peoples' contributions have gone up from £975 to £1,169. The latter is a hopeful sign, and if the working classes continue to give more to the Infirmary they may feel sure that the richer people will follow suit. The total number of in-patients was 883. There was a daily average number resident of 53, and the average duration of each patient's stay in the hospital was 27 days. The medical and surgical tables give sufficient information. There were 32 cases of pneumonia, of which 28 were cured and 2 died. Of 21 cases of rheumatic fever, 16 recovered, 4 were relieved, and 1 died. A total of 41 amputations show as results 38 cured, 2 relieved, and 1 death. The total number of operations was 275, of which 248 were successful, 20 partly successful, 4 were followed by death, and 3 remained in the hospital. We regret that the house surgeon has not favoured us with an anæsthetic table; and the Committee tell us nothing about the average cost per patient or per bed occupied, or the cost per head per week, information which we hope will be supplied in the next report.

### GENERAL HOSPITAL, BIRMINGHAM.

(STATISTICAL REPORT.)

ON the first of January this hospital contained 291 patients, and 4,650 were admitted during the year, making a total of 4,941 under treatment. Of this total 2,917 were discharged cured, 905 relieved, 155 were incurable, 216 were discharged to the Jaffray Branch Hospital, 481 died, and 267 remained in the wards. The out-patients numbered 64,926. The medical and surgical tables are divided into columns, showing the numbers cured, relieved, not relieved, died, and the totals. We notice that 45 cases of rheumatic fever yielded 37 recoveries and 8 relieved. There were 83 cases of acute lobar pneumonia, and of these 65 recovered, 7 were relieved, and 11 died. The surgical operations are given in detail. The disease is in every instance named, the operation performed, the sex and age of the patients, and the date of the operation, and the date of discharge. There is a column for remarks and the operating surgeon's name is given. The anæsthetic table shows that the total number of patients to whom anæsthetics were given was 2,824. Ether alone, or in combination with ethyl chloride, or with nitrous oxide, was the agent in 495 cases, chloroform in 778, ethyl chloride in 505, nitrous oxide, ethyl chloride and chloroform in 34, other sequences and mixtures of ether and chloroform in 296, nitrous oxide in 245, and ethyl chloride in 471. Five deaths are recorded as having taken place during anæsthesia. One was during the administration of nitrous oxide in the case of a man with enlarged glands in the neck and the left pleura adherent. The agent was given for the extraction of teeth. There was no dyspnœa, and nothing noteworthy took place during the inhalation until the respiration and circulation failed. The other deaths took place under chloroform. One in a girl of 17 when a large double-sided goitre was being removed. Another in a child of 6 for examination of chest and abdomen. The chest was found to be full of fluid. The third was a child of 4 with intense dyspnœa due to diphtheria. The fourth was an alcoholic man of 41. He was on the verge of delirium tremens, and the post-mortem examination revealed chronic inflammation of the brain, fatty heart, bronchitic lungs and adherent pleura. There were a good many cases of pneumonia and bronchitis consequent on the inhalation of ether. In the $x$-ray and light department 81 cases of lupus, rodent ulcer, cancer, etc., have been treated, and it is stated that over 2,500 applications of light and $x$-rays have been administered with satisfactory results.

